# The effect of baseline pressure errors on an intracranial pressure-derived index: results of a prospective observational study

**DOI:** 10.1186/1475-925X-13-99

**Published:** 2014-07-23

**Authors:** Per Kristian Eide, Angelika Sorteberg, Torstein R Meling, Wilhelm Sorteberg

**Affiliations:** 1 Department of Neurosurgery, Oslo University Hospital, Rikshospitalet, Oslo, Norway; 2 Faculty of Medicine, University of Oslo, Oslo, Norway

## Abstract

**Background:**

In order to characterize the intracranial pressure-volume reserve capacity, the correlation coefficient (R) between the ICP wave amplitude (A) and the mean ICP level (P), the RAP index, has been used to improve the diagnostic value of ICP monitoring. Baseline pressure errors (BPEs), caused by spontaneous shifts or drifts in baseline pressure, cause erroneous readings of mean ICP. Consequently, BPEs could also affect ICP indices such as the RAP where in the mean ICP is incorporated.

**Methods:**

A prospective, observational study was carried out on patients with aneurysmal subarachnoid hemorrhage (aSAH) undergoing ICP monitoring as part of their surveillance. Via the same burr hole in the scull, two separate ICP sensors were placed close to each other. For each consecutive 6-sec time window, the dynamic mean ICP wave amplitude (MWA; measure of the amplitude of the single pressure waves) and the static mean ICP, were computed. The RAP index was computed as the Pearson correlation coefficient between the MWA and the mean ICP for 40 6-sec time windows, i.e. every subsequent 4-min period (method 1). We compared this approach with a method of calculating RAP using a 4-min moving window updated every 6 seconds (method 2).

**Results:**

The study included 16 aSAH patients. We compared 43,653 4-min RAP observations of signals 1 and 2 (method 1), and 1,727,000 6-sec RAP observations (method 2). The two methods of calculating RAP produced similar results. Differences in RAP ≥0.4 in at least 7% of observations were seen in 5/16 (31%) patients. Moreover, the combination of a RAP of ≥0.6 in one signal and <0.6 in the other was seen in ≥13% of RAP-observations in 4/16 (25%) patients, and in ≥8% in another 4/16 (25%) patients. The frequency of differences in RAP >0.2 was significantly associated with the frequency of BPEs (5 mmHg ≤ BPE <10 mmHg).

**Conclusions:**

Simultaneous monitoring from two separate, close-by ICP sensors reveals significant differences in RAP that correspond to the occurrence of BPEs. As differences in RAP are of magnitudes that may alter patient management, we do not advocate the use of RAP in the management of neurosurgical patients.

## Background

Monitoring of intracranial pressure (ICP) is crucial in the intensive care management of neurosurgical patients [1-4]. The common management goal is to maintain the static pressure parameter mean ICP <20-25 mmHg [3]. When the cardiac-induced ICP waves are monitored as well, the goal is to keep the dynamic pressure parameter mean ICP wave amplitude (MWA) <5 mmHg [1]. In an attempt to better characterize the intracranial pressure-volume reserve capacity, some authors have also calculated an index measuring the correlation coefficient (R) between the ICP wave amplitude (A) and the mean ICP level (P), denoted RAP [5,6]. Repetitive computation of this RAP index has been used in the surveillance of patients with traumatic brain injury (TBI), cerebral bleeds, and hydrocephalus [6-9]. Using the RAP index, which ranges from -1 to +1, the upper normal threshold is +0.6 [6,8-12]. A RAP approaching +1 has hence been considered as indicative of reduced compensatory reserve capacity, or impaired intracranial compliance.

The mean ICP is always determined against a baseline pressure (i.e. zero or reference pressure). A major weakness of this is that the mean ICP value that is displayed to the physician or nurse becomes erroneous if this baseline pressure is spontaneously altered during ongoing monitoring [13]. Such baseline pressure errors (BPEs) occur during clinical ICP monitoring, and has been seen in a variety of ICP sensor types and locations from where the ICP is recorded [13,14]. In contrast, the dynamic ICP parameters, such as the MWA remain unaffected by BPEs as they express just changes of ICP within each heartbeat.

In a recent prospective, observational study on 16 patients with aneurysmal subarachnoid haemorrhage (SAH), we reported a high frequency and severity of BPEs [15]. Being recognized as differences in mean ICP in combination with close to identical MWA, BPEs affect the RAP index, causing the RAP to differ widely when determined from two separate ICP sensors in the same patient [16]. This is most evident when monitoring ICP from two different types of ICP sensors [16]. The present study was undertaken to explore the frequency and severity of differences in RAP when using two ICP sensors of the same type. To this end, we reanalysed the continuous ICP recordings of 16 aSAH patients enrolled in a prospective, observational study in which we recorded the ICP from two separate ICP sensors placed in the brain tissue via the same burr hole in the scull [15].

## Methods

### Patients

Aneurysmal SAH patients who need continuous ICP monitoring as part of their intensive care management were enrolled in the study. Their intensive care management was not influenced by their participation.

The study was approved by The Regional Ethics Committee, REK South-East (2010/1328B) and Oslo University Hospital (2010/16315), Oslo, Norway. Inclusion was by oral and written informed consent, either by the patient herself/himself or by the closest family member.

### Study design

A prospective, observational study design was used to determine the frequency and severity of BPEs, and how BPEs affect scores of ICP and ICP indices. These data were re-analyzed in order to investigate the frequency and severity of differences in RAP. Moreover, we compared two different methods of calculating the RAP.

### Monitoring and analysis of ICP

Two Raumedic NeuroVent P (Raumedic AG, Münchberg, GE) ICP sensors were placed in the brain parenchyma via the same burr hole, either during aneurysm surgery, or during placement of an external ventricular drain (EVD). The location of the ICP sensors was verified postoperatively by cerebral computer tomography (CT) scanning.

Both sensors were connected to the MPR-1 monitor (Raumedic AG, Münchberg, GE), which in turn was connected to a laptop computer running Sensometrics Software (dPCom AS, Oslo, Norway). The digital sampling rate of pressure signals from the two sensors (Sensors 1 and 2) was 100 Hz. The raw data files were stored on the computer. Recordings from the two sensors continued throughout the time period the patient was in need of ICP surveillance.

**Figure 1:**
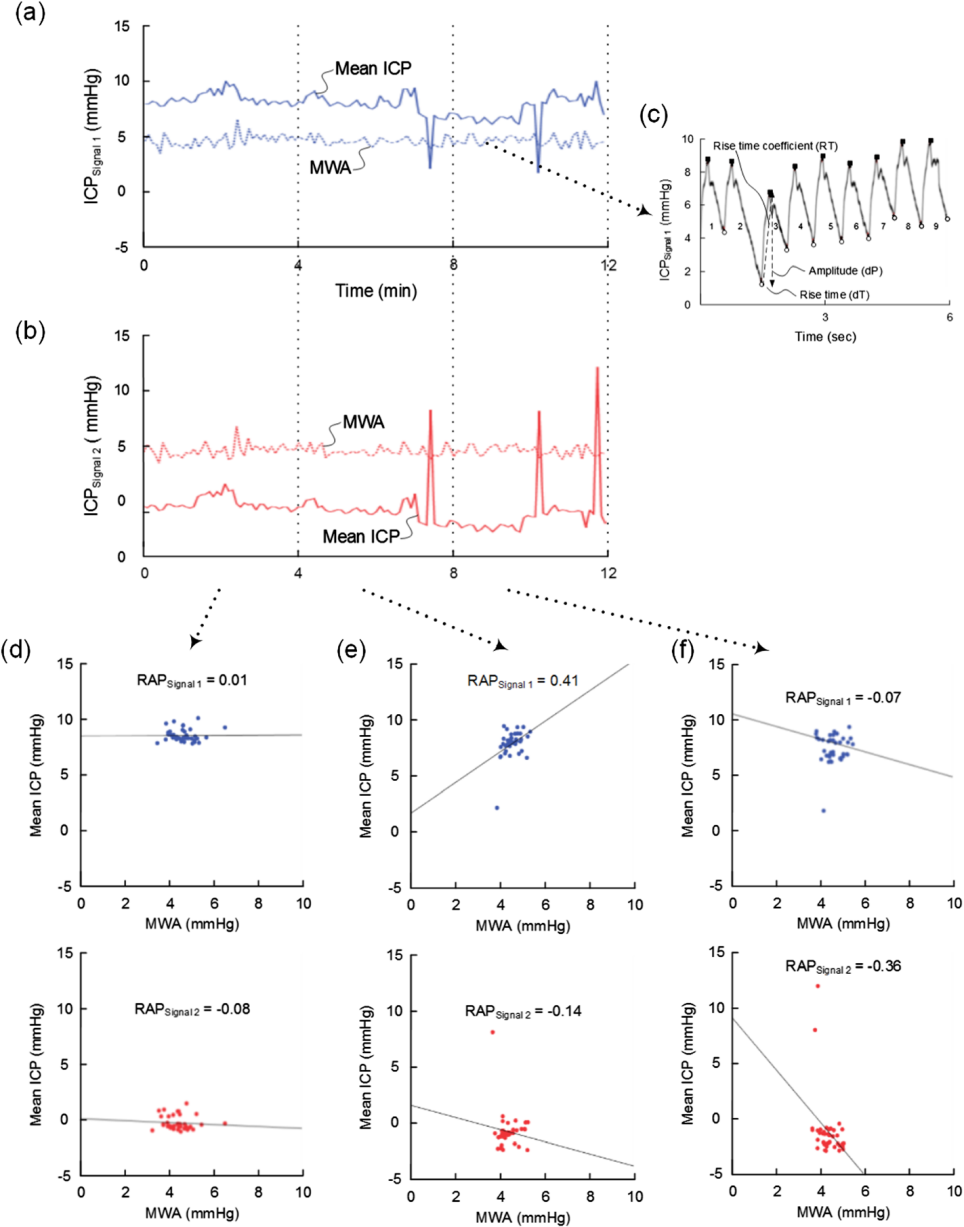
**Illustration of the method of determining the RAP [correlation coefficient (R) between the intracranial pressure (ICP) wave amplitude (A) and the mean ICP level (P)] in patient 2.** Following automatic identification of the cardiac induced single intracranial pressure (ICP) waves, the mean ICP and mean wave amplitude (MWA) are determined for every consecutive 6-sec time window. Trend plots of mean ICP and MWA determined during the same 6-sec time windows are shown for Signals 1 **(a)** and 2 **(b)** over subsequent 12 min periods (representing three 4-min periods, and 120 6-sec time windows). For Signal 1 **(a)** the average (± standard deviation) of mean ICP was 7.9 ± 1.2 mmHg and of MWA 4.6 ± 0.5 mmHg; while for Signal 2 **(b)** mean ICP -0.9 ± 1.9 mmHg and MWA 4.4 + 0.5 mmHg (mean difference of ICP -8.8 ± 2.2 mmHg; mean difference of MWA -0.2 ± 0.2 mmHg). In **(c)** one single 6-sec time window is shown, demonstrating the individual single ICP waves, each wave being characterized by the amplitude (dP), rise time (dT), and rise time coefficient (RT) (indicated for single wave 3). RAP is determined as the Pearson correlation coefficient between MWA and the mean ICP during subsequent 4 min periods (representing 40 6-sec time windows). In the patients included in this study, we compared the RAP values during identical 4-min periods for Signals 1 and 2 (referred to as method 1). This was performed for every consecutive 4-min period. For the three consecutive 4-min periods shown in **(a)** and **(b)**, the corresponding scatter plots and RAP-values are presented in **(d)**, **(e)**, and **(f)**, respectively. The differences in RAP were associated with marked differences in mean ICP between Signals 1 and 2 whereas the MWA was close to identical.

As previously described, the ICP signals were analyzed according to the methodology implemented in Sensometrics Software (Figure 1) [17]. An automatic method identifies the heartbeat-induced single ICP waves, differentiating them from the pressure waves of other origins (noise or various artifacts). Each heartbeat-induced single ICP wave is characterized by the following wave parameters: the amplitude (dP), rise time (dT), and the rise-time coefficient (dP/dT) (Figure 1c). Only 6-sec time windows containing a minimum of four cardiac-beat-induced waves were included for further analysis. The MWA is computed in consecutive 6-sec time window (Figure 1c). During the same 6-sec time windows, the mean ICP is determined as the sum of sample values divided by the number of samples.

### Calculation of RAP

**Figure 2:**
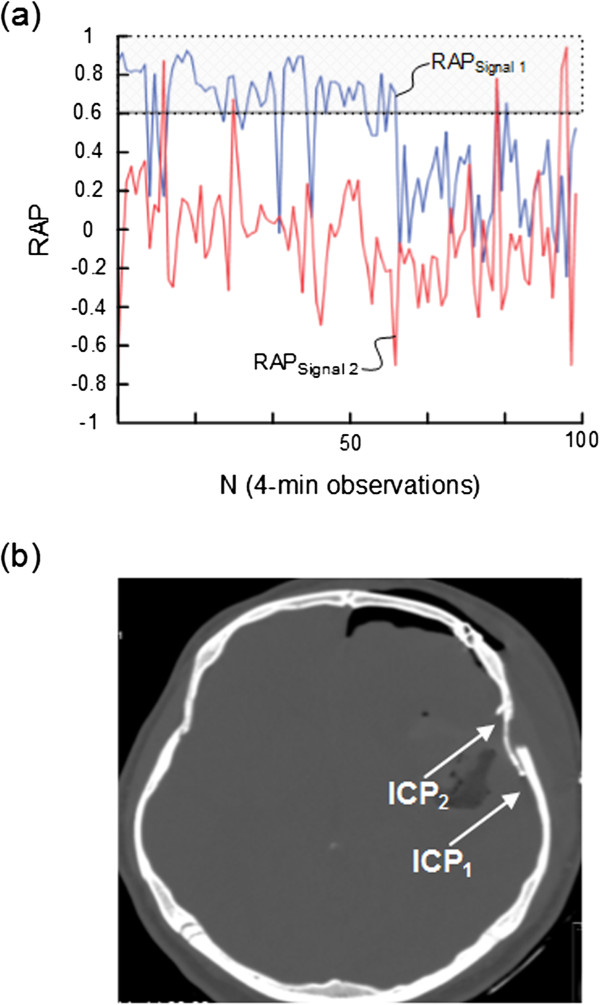
**Trend plots of RAP [correlation coefficient (R) between the intracranial pressure (ICP) wave amplitude (A) and the mean ICP level (P)] of Signals 1 and 2 in patient 2.** For patient 2, the trend plots of **(a)** RAP determined during 100 consecutive 4-min periods for signals 1 (blue line) and 2 (red line) show marked differences (average of RAP_Signal 1_ 0.50; average of RAP_Signal 2_ -0.04). The horizontal lines at RAP 0.6 illustrate a commonly used upper normal threshold for RAP. The intracranial locations of the ICP sensors 1 and 2 are illustrated in **(b)**.

The software incorporates an automatic procedure for determining the correlation coefficient (R) between the ICP wave amplitude (A) and the ICP level (P), the RAP, during consecutive 4-min time periods. The RAP-index is the Pearson correlation coefficient between the MWA and the mean ICP during 40 6-sec time window periods. Computation of RAP has previously been described by others [6,12]. Since we compared the RAP of two simultaneous ICP signals, the RAP of Sensors 1 and 2 were derived from simultaneous 6-sec time windows (Figures 1a-b).The Pearson correlation coefficient is a measure of the strength of a relationship between two variables, ranging from -1 to +1. When one variable changes in the opposite direction of the other, the correlation coefficient becomes negative, whereas the correlation coefficient becomes positive when both variables change in the same direction (Figures 1d-f). The closer the correlation coefficient is to + or -1, the stronger is the relationship between the two variables. The assumptions for using the Pearson correlation coefficient during 4-min periods as performed in this study were fulfilled: Both the mean ICP and the MWA are continuous and independent observations that follow a normal distribution. Moreover, for intervals of 4-minute duration, the correlation coefficient between these two observations reflects a linear relationship.We further compared two different methods of calculating the RAP:(i) Method 1. According to method 1, a new RAP value was calculated every 4 min period. Hence, for every consecutive 4-min period the software determined the Pearson correlation coefficient (RAP) values of the two ICP signals (Figures 1c-d). The RAP scores could then be trended as shown in Figure 2.

(ii) Method 2. This method incorporates a moving time window. A new RAP value is updated every 6 second. Consequently, every 6 second, the ‘oldest’ averaged value drops out and the ‘newest’ drops in. This approach was previously described by others [18], except that in this latter study the authors used 5 min intervals and 10 seconds windows, as compared to 4 min intervals and 6 seconds windows in the present paper.

### Determination of baseline pressure errors (BPEs)

BPEs were defined as differences in mean ICP in combination with close to identical MWAs (difference between sensors <0.5 mmHg), as recently described in this journal [15]. In this study, we determined the percentage of recording time with BPEs of given magnitudes (5 mmHg ≤ BPE < 10 mmHg) for every patient.

## Results

### Patients

The study enrolled a total of 16 aSAH patients, 8 females and 8 males, with median age 58 (ranges 39 – 74) years (Table 1, left). The distances between the two ICP sensors are presented in Table 1, right and were: axially median 11 (ranges 2-38) mm, coronally median 8 (ranges 3-20) mm and sagitally median 9 (ranges 3-26) mm. No adverse effects of the ICP monitoring were observed.

**Table 1:** Demographic data of the 16 patients included in the study, and distance between Sensors 1 and 2

**Pat**	**Age**	**Gender**	**Distance between ICP Sensors 1 and 2 (mm)**		
			**Axial**	**Coronal**	**Sagittal**
1	52	M	13	9	7
2	57	M	30	17	22
3	64	M	6	17	11
4	59	F	3	5	11
5	58	F	38	13	25
6	39	F	13	11	7
7	58	F	11	6	18
8	66	M	4	5	5
9	44	M	3	3	3
10	70	M	7	4	5
11	49	M	11	5	6
12	52	F	21	20	26
13	63	F	2	4	4
14	74	F	13	11	11
15	56	F	3	3	9
16	55	M	19	19	8
Median (Ranges)	58 (39-74)	8 F – 8 M	11 (2-38)	8 (3 - 20)	9 (3 - 26)

### Method 1: comparison of 4-min RAP-values between signals 1 and 2

Table 2 left shows the number of 4-min RAP observations obtained in the 16 patients. For all patients combined, 43,653 4-min RAP-values were available for analysis; median number for the patients was 2,472 (ranges 1,090 – 5,522).Figure 1 illustrates how differences in RAP between Signals 1 and 2 were associated with marked differences in mean ICP whereas the MWAs remained close to identical during the recording.

**Table 2:** Comparison of 4-min RAP-values between Signals 1 and 2

**PatID**	**N (4-min RAP observations)**	**RAP (average + std)**		**Differences in RAP between Signals 1 and 2 (N,%)**		
		**Signal 1**	**Signal 2**	**0.2**	**0.4**	**0.6**
1	1,850	0.23 ± 0.43	0.26 ± 0.42	483 (26%)	147 (8%)	51 (3%)
2	1,090	0.15 ± 0.36	-0.01 ± 0.36	612 (56%)	338 (31%)	179 (16%)
3	4,706	0.61 ± 0.33	0.62 ± 0.32	365 (8%)	72 (2%)	30 (1%)
4	2,119	0.36 ± 0.38	0.36 ± 0.38	200 (9%)	29 (1%)	8
5	1,651	0.60 ± 0.35	0.61 ± 0.35	53 (3%)	11 (1%)	3
6	1,119	0.42 ± 0.35	0.43 ± 0.35	187 (17%)	54 (5%)	10 (1%)
7	3,209	0.65 ± 0.30	0.68 ± 0.28	236 (7%)	83 (3%)	35 (1%)
8	3,747	0.51 ± 0.39	0.35 ± 0.41	1,311 (35%)	806 (22%)	456 (12%)
9	4,221	0.53 ± 0.40	0.61 ± 0.34	982 (23%)	506 (12%)	323 (8%)
10	3,538	0.25 ± 0.33	0.26 ± 0.33	166 (5%)	20 (1%)	11
11	1,494	0.62 ± 0.32	0.63 ± 0.31	54 (4%)	14 (1%)	4
12	3,089	0.83 ± 0.24	0.83 ± 0.25	19 (1%)	3	2
13	1,381	0.28 ± 0.34	0.32 ± 0.35	413 (30%)	113 (8%)	26 (2%)
14	2,093	0.38 ± 0.34	0.37 ± 0.35	254 (12%)	72 (3%)	31 (1%)
15	5,522	0.52 ± 0.36	0.50 ± 0.37	729 (13%)	142 (3%)	28 (1%)
16	2,824	0.38 ± 0.36	0.39 ± 0.36	393 (14%)	73 (3%)	15 (1%)

RAP: correlation coefficient (R) between the intracranial pressure (ICP) wave amplitude (A) and the mean ICP level (P).

**Figure 3:**
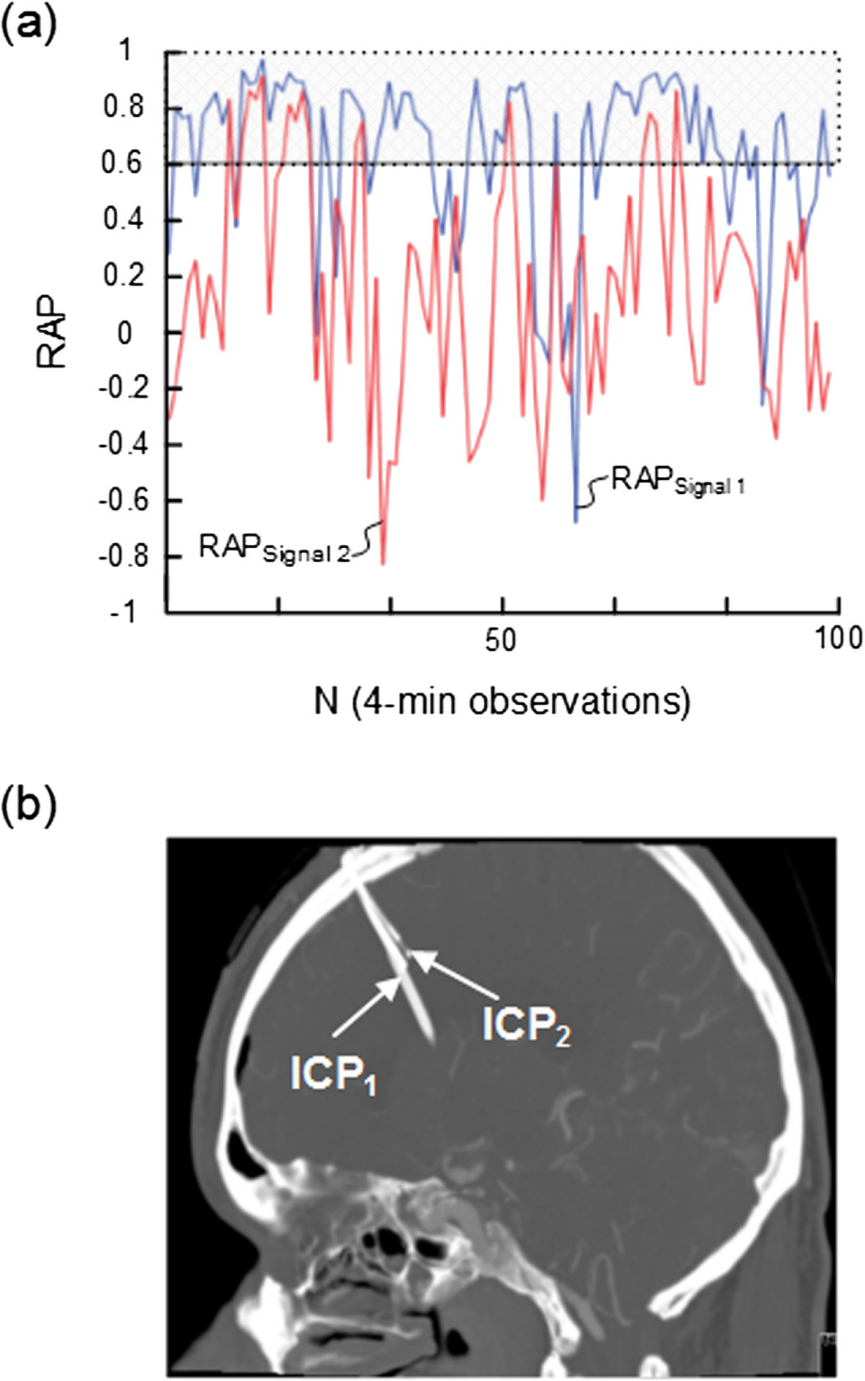
**Trend plots of RAP [correlation coefficient (R) between the intracranial pressure (ICP) wave amplitude (A) and the mean ICP level (P)] of Signals 1 and 2 in patient 8.** For patient 8 the trend plots of **(a)** RAP determined during 100 consecutive 4-min periods for signals 1 (blue line) and 2 (red line) show marked differences (average of RAP_Signal 1_ 0.64; average of RAP_Signal 2_ 0.16). The horizontal lines at RAP 0.6 illustrate a commonly used upper normal threshold for RAP. The intracranial locations of the ICP sensors 1 and 2 are shown in **(b)**.

**Figure 4:**
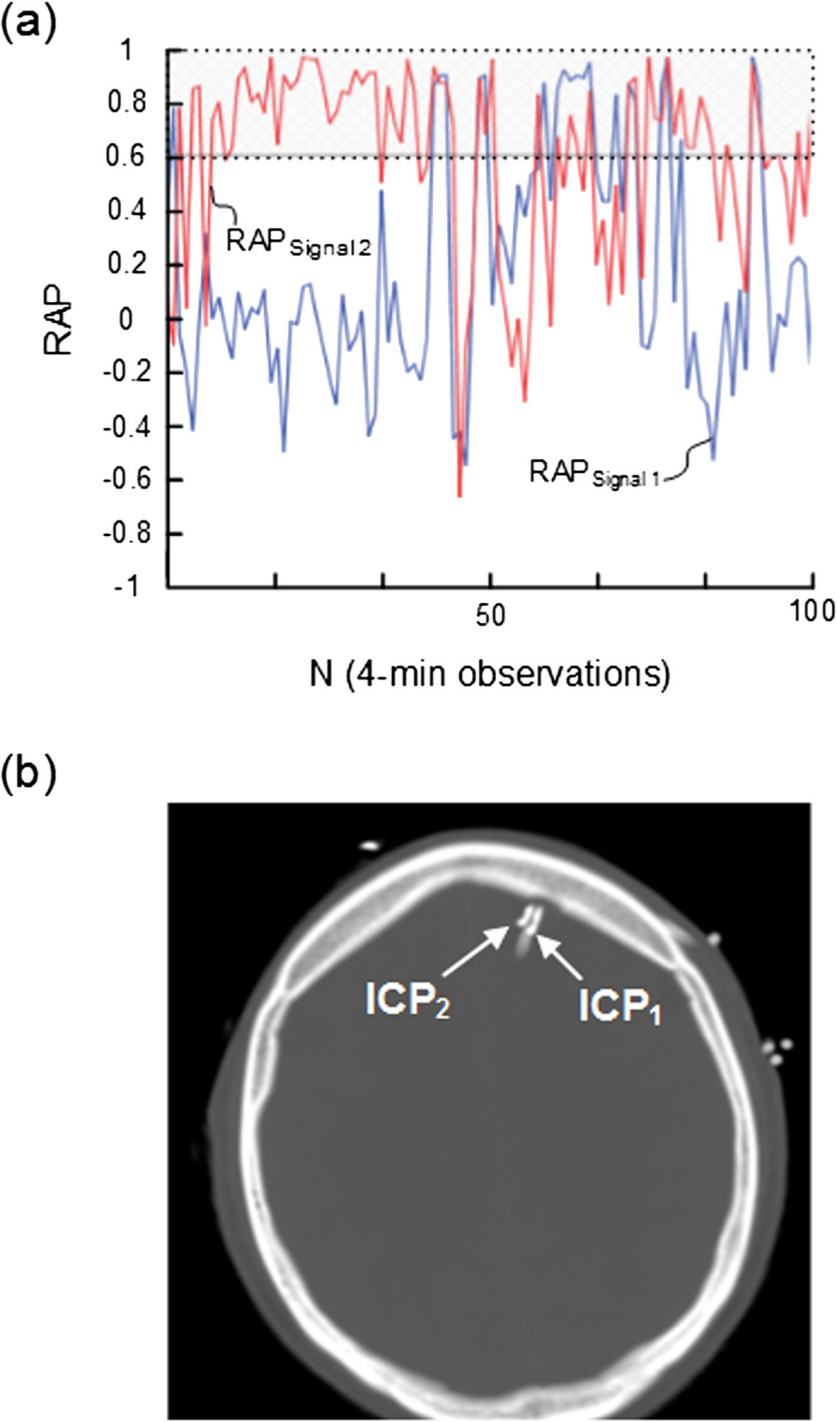
**Trend plots of RAP [correlation coefficient (R) between the intracranial pressure (ICP) wave amplitude (A) and the mean ICP level (P)] of Signals 1 and 2 in patient 9.** For patient 9 the trend plots of **(a)** RAP determined during 100 consecutive 4-min periods for signals 1 (blue line) and 2 (red line) show marked differences (average of RAP_Signal 1_ 0.17; average of RAP_Signal 2_ 0.59). The horizontal lines at RAP 0.6 illustrate a commonly used upper normal threshold for RAP. The intracranial locations of the ICP sensors 1 and 2 are shown in **(b)**.

Simultaneous 4-min RAP scores (determined according to method 1) from Signal 1 and Signal 2 are presented in Table 2. While Table 2, middle, lists the RAP scores (mean and ± standard deviation) for each patient, Table 2, right presents differences in RAP between the two signals that were ≥ 0.2, ≥0.4, and ≥0.6, respectively. Major differences (≥0.4) in RAP were seen in 5 (31%) of 16 patients, including patients 1, 2, 8, 9, and 13 (Table 2, right). The trend plots of RAP of the two signals are visualized for subjects 2, 8, and 9 in Figures 2a, 3a, and 4a, respectively. The locations of the ICP sensors for these three patients are shown on CT scans in Figures 2b, 3b, and 4b, respectively.

Table 3 presents the portion of 4-min observations wherein RAP was <0.6 in both signals (left), ≥0.6 in both signals (middle) and <0.6 in one signal and ≥0.6 in the other (right). The combination of a RAP of <0.6 in one signal and ≥0.6 in the other was seen in ≥14% of scores in 4/16 (25%) patients (patients 2, 8, 9 and 13), and in ≥9% of observations in another 4/16 (25%) patients) (patients 3, 6, 7, and 15).

**Table 3:** Proportion of 4-min observations with RAP <0.6 in both signals (left), RAP ≥ 0.6 in both signals (middle) and RAP ≥ 0.6 in one signal while RAP < 0.6 in another signal (right)

**PatID**	**RAP**_**SIGNAL 1**_ **< 0.6/RAP**_**SIGNAL 2**_ **< 0.6**	**RAP**_**SIGNAL 1**_ **≥ 0.6/RAP**_**SIGNAL 2**_ **≥ 0.6**	**RAP**_**SIGNAL 1**_ **≥ 0.6/RAP**_**SIGNAL 2**_ **< 0.6 or RAP**_**SIGNAL 1**_ **< 0.6/RAP**_**SIGNAL 2**_ **≥ 0.6**

1	1,360 (73.5%)	369 (20%)	121 (6.5%)
2	910 (83.5%)	19 (1.7%)	161 (14.8%)
3	1,521 (32%)	2,776 (59%)	409 (9%)
4	1,371 (64.7%)	624 (29.4%)	124 (5.9%)
5	547 (33.1%)	1,017 (61.6%)	87 (5.3%)
6	652 (58%)	368 (33%)	99 (9%)
7	779 (24%)	2,148 (67%)	282 (9%)
8	45.6 (45%)	1,231 (32.9%)	809 (21.6%)
9	1,498 (35.5%)	2,051 (48.6%)	672 (15.9%)
10	2,800 (79.1%)	598 (16.9%)	140 (4%)
11	521 (34.9%)	891 (59.6%)	82 (5.5%)
12	323 (10.5%)	2,719 (88.0%)	47 (1.5%)
13	974 (70%)	217 (16%)	190 (14%)
14	1,349 (64.5%)	585 (28%)	159 (7.6%)
15	2,619 (47%)	2,425 (44%)	478 (9%)
16	1,798 (63.7%)	830 (29.4%)	196 (6.9%)

RAP: correlation coefficient (R) between the intracranial pressure (ICP) wave amplitude (A) and the mean ICP level (P).

### Method 2: comparison of 6-sec RAP-values between signals 1 and 2

**Figure 5:**
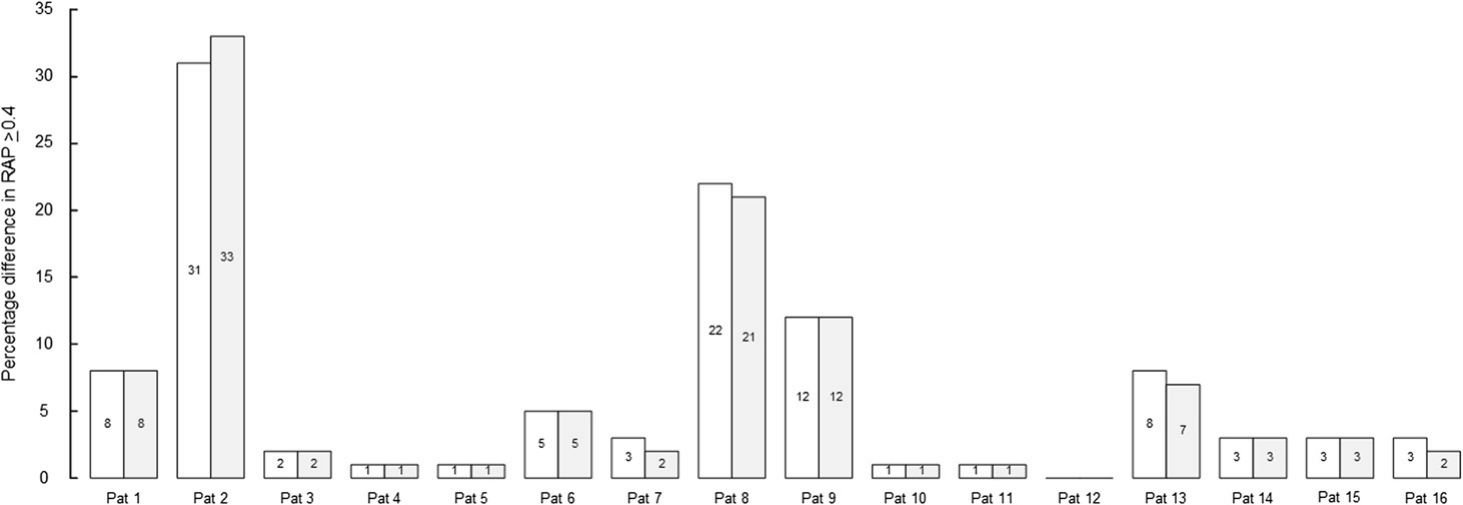
**Comparisons of methods 1 and 2 regarding percentage of observations with differences in RAP** ≥**0.4 between signals 1 and 2.** The percentages of RAP-differences ≥0.4 between signals 1 and 2 were determined for each patient using methods 1 (open boxes) and 2 (grey-shaded boxes). The percentage for each individual is given within each box. The values are also given in Table 2 (Method 1) and 4 (Method 2).

The 6-sec RAP observations of the 16 patients are shown in Table 4. A total of 1,727,000 6-sec RAP values were analyzed; median number for the 16 patients was 97,922 (ranges 42,162 – 220,276). Table 4, right shows differences in RAP between the two signals that were ≥0.2, ≥0.4, and ≥0.6, respectively. Major differences (≥0.4) in RAP were seen in 5 of 16 patients (31%), including patients 1, 2, 8, 9, and 13 (Table 4, right). Figure 5 illustrates that computation of RAP according to methods 1 and 2 gave close to identical results, as illustrated by the percentage of differences in RAP between signals 1 and 2 ≥ 0.4.

**Table 4:** Differences in 6-sec RAP-values between Signals 1 and 2

**Pat ID**	**N (6-sec RAP observations)**	**Differences in RAP between Signals 1 and 2 (N,%)**		
		**0.2**	**0.4**	**0.6**
1	73,005	18,935 (26%)	5,907 (8%)	1,981 (3%)
2	42,162	24,602 (58%)	13,710 (33%)	7,173 (17%)
3	206,708	17,604 (9%)	4,773 (2%)	1,793 (1%)
4	78,874	6,387 (8%)	938 (1%)	255
5	43,936	1,162 (3%)	250 (1%)	81
6	43,888	8,145 (19%)	2,288 (5%)	727 (2%)
7	126,768	8,545 (7%)	2,939 (2%)	1,205 (1%)
8	149,625	52,031 (35%)	31,938 (21%)	17,830 (12%)
9	167,244	37,871 (23%)	19,370 (12%)	11,763 (7%)
10	141,358	6,485 (5%)	1,015 (1%)	345
11	59,520	2,205 (4%)	527 (1%)	161
12	123,382	742 (1%)	127	57
13	54,411	15,663 (29%)	4,065 (7%)	1,069 (2%)
14	83,498	9,689 (12%)	2,793 (3%)	1,142 (1%)
15	220,276	31,022 (14%)	7,126 (3%)	1,677 (1%)
16	112,345	14,299 (13%)	2,657 (2%)	601 (1%)

RAP: correlation coefficient (R) between the intracranial pressure (ICP) wave amplitude (A) and the mean ICP level (P).

**Figure 6:**
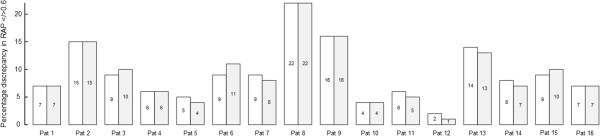
**Comparisons of methods 1 and 2 regarding percentage of observations with discrepancy in RAP </**≥**0.6 between signals 1 and 2.** The percentages of observations wherein one signal showed RAP ≥0.6 while the other showed RAP <0.6 were determined for each patient using methods 1 (open boxes) and 2 (grey-shaded boxes). The percentage for each individual is given within each box. The values are also given in Table 3 (Method 1) and 5 (Method 2).

Table 5 presents the portion of 6-sec observations wherein RAP was <0.6 in both signals (left), ≥0.6 in both signals (middle) and <0.6 in one signal and ≥0.6 in the other (right). The combination of a RAP of <0.6 in one signal and ≥0.6 in the other in ≥8% of observations was seen 8/16 (50%) patients (patients 2, 3, 6-9, 13 and 15). Figure 6 further illustrates that the two different methods of calculating the RAP gave similar results regarding the percentage of observations where RAP was <0.6 in one signal and ≥0.6 in the other.

**Table 5:** Proportion of 6-sec observations of RAP <0.6 in both signals (left), RAP ≥ 0.6 in both signals (middle) and RAP ≥ 0.6 in one signal while RAP < 0.6 in another signal (right)

**Pat ID**	**RAP**_**SIGNAL 1**_ **< 0.6/RAP**_**SIGNAL 2**_ **< 0.6**	**RAP**_**SIGNAL 1**_ **≥ 0.6/RAP**_**SIGNAL 2**_ **≥ 0.6**	**RAP**_**SIGNAL 1**_ **≥ 0.6/RAP**_**SIGNAL 2**_ **< 0.6 or RAP**_**SIGNAL 1**_ **< 0.6/RAP**_**SIGNAL 2**_ **≥ 0.6**

1	53,345 (73.1%)	14,269 (19.5%)	5,391 (7.4%)
2	35,400 (84.0%)	498 (1.2%)	6,264 (14.9%)
3	71,434 (34.6%)	114,056 (55.2%)	21,218 (10.3%)
4	51,401 (65.2%)	22,404 (28.4%)	5,069 (6.4%)
5	12,803 (29.1%)	29,420 (67.0%)	1,713 (3.9%)
6	25,215 (57.5%)	13,893 (31.7%)	4,780 (10.9%)
7	31,849 (25.1%)	85,127 (67.2%)	9,792 (7.7%)
8	69,759 (46.6%)	47,354 (31.6%)	32,512 (21.7%)
9	60,638 (36.3%)	80,763 (48.3%)	25,843 (15.5%)
10	112,598 (79.7%)	23,769 (16.8%)	4,991 (3.5%)
11	21,155 (35.5%)	35,341 (59.4%)	3,024 (5.1%)
12	12,949 (10.5%)	108,783 (88.2%)	1,650 (1.3%)
13	38,716 (71.2%)	8,457 (15.5%)	7,238 (13.3%)
14	54,390 (65.1%)	23,122 (27.7%)	5,986 (7.2%)
15	104,728 (47.5%)	94,725 (43.0%)	20,823 (9.5%)
16	71,439 (63.6%)	33,122 (29.5%)	7,784 (6.9%)

RAP: correlation coefficient (R) between the intracranial pressure (ICP) wave amplitude (A) and the mean ICP level (P).

### BPEs of various magnitudes versus difference in RAP

**Figure 7:**
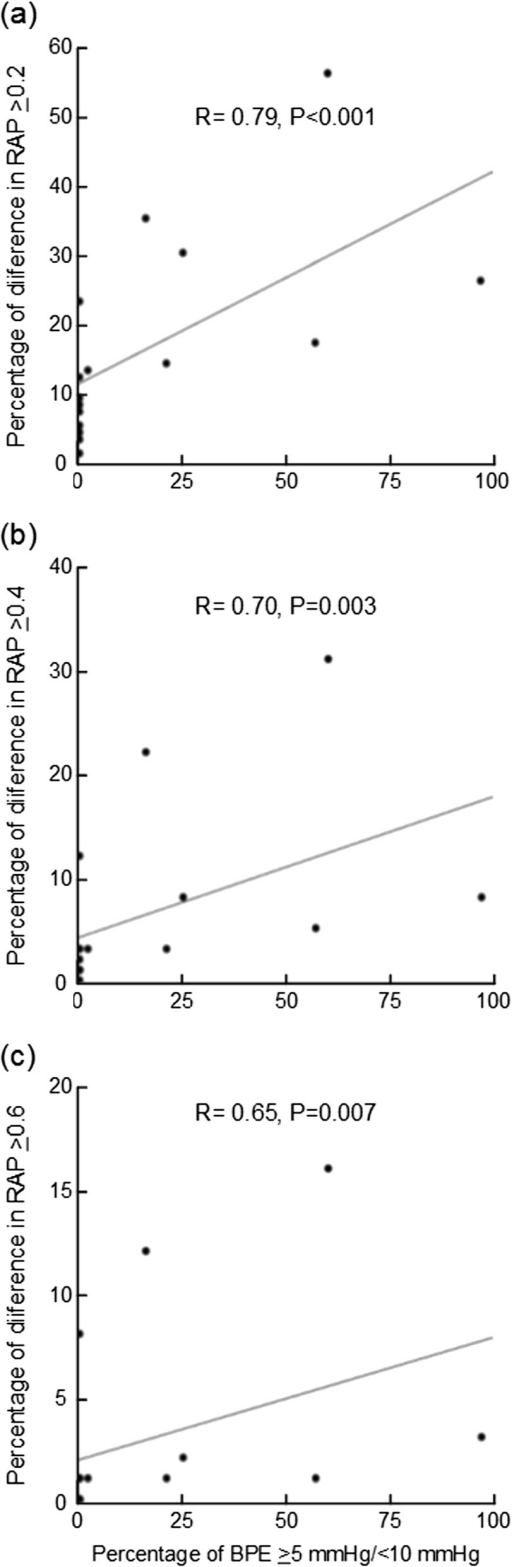
**Correlation plots of percentages of baseline pressure errors (BPEs) and RAP [correlation coefficient (R) between the intracranial pressure (ICP) wave amplitude (A) and the mean ICP level (P)]-differences.** The percentages of RAP-differences are plotted against the percentages of BPEs of various magnitudes for the 16 patients of the study. The percentages of BPEs of magnitudes ≥5 mmHg/<10 mmHg were plotted against differences in RAP of **(a)** ≥0.2 or **(b)** ≥0.4, and **(c)** ≥0.6. The Spearman correlation coefficients are given suggesting significant correlation between percentages of BPEs and percentages of RAP-differences. The plots were created based on percentages provided in Table 4.

Figure 7 presents the correlation between percentages of BPEs of given magnitude (5 mmHg ≤ BPE < 10 mmHg) and the percentage of 4-min periods with RAP difference being ≥0.2 (Figure 7a), ≥0.4 (Figure 7b), and ≥0.6 (Figure 7c). The correlation plots demonstrate that in patient recordings with a high percentage of BPEs, there is also a high percentage of marked differences in RAP, i.e. the occurrences of differences in RAP are associated with the occurrences of BPEs.

## Discussion

The main observation of this study was a marked difference in RAP indices [moving correlation coefficient (R) between the ICP wave amplitude (A) and the ICP (P)] obtained from simultaneous registrations from two separate, close-by ICP sensors of the same type. The discrepancy in RAP between signals 1 and 2 was independent of the use of a moving window with frequent updates. The occurrences of differences in RAP were associated with occurrences of baseline pressure errors (BPEs). While the number of patients was rather small (n = 16), the number of RAP observations was high (total 43,653 4-min RAP observations and 1,727,000 6-sec RAP observations), with a median number of RAP-observations for each patient (median 2,472).

### Clinical use of ICP-derived indices

ICP derived indices were introduced in the early 1990’s to enhance the diagnostic information of ICP monitoring [6,12]. In particular, the RAP index has been used as a possible indicator of the intracranial pressure volume compensatory reserve capacity [5,7,10,12]. Several authors have found the RAP to be of value in the surveillance of patients with TBI [6,9,10,12,19] and in the diagnostic assessment of hydrocephalus [7,8,11]; then proposing an upper normal threshold value of about +0.6 [6,8-11]. However, the clinical usefulness of this index remains to be determined [20-22].

In contrast, as compared to RAP scores derived from amplitudes computed using the frequency domain method [6,12], we presently used an amplitude computed from the time domain method (the MWA) [17]. The frequency- and time-domain methods for calculating single pressure wave amplitudes are not equivalent [23]. The time domain method has the advantage of correctly identifying the heartbeat-induced pressure waves versus artifact waves. Using the frequency domain method, on the other hand, the amplitude is retrieved from the first harmonic of the power spectrum, hence providing an approximation of the amplitude [23]. In this present work, which compared RAP values, the mode of amplitude computation should not affect the results.

It can be discussed how many data points that should be used for computation of RAP. Most recent studies have determined RAP from 40 data points separated by 6 seconds over 4 min periods [3,7-9,11]. We used the same approach in this present study.

### Comparison of RAP scores from two simultaneous ICP signals

Combining the present RAP findings with those of an earlier study [16], differences in RAP are smaller when comparing signals from similar ICP sensor types and larger when comparing signals from different ICP sensor types [16]. The largest differences were hence seen when monitoring from one solid - and one fluid ICP sensor.

The present findings extend our previous observations of marked differences in 4-min RAP when monitored simultaneously from two separate ICP sensors [16]. A difference in 4-min RAP ≥0.4 was hence seen in ≥8% of observations in 5/16 (31%) patients. Determining the proportion of 4-min observations wherein the RAP was ≥0.6 in one signal and <0.6 in another (above normal threshold level in one sensor and below in the other) revealed this setting in >14% of scores in 4/16 (25%) patients and >9% of scores in another 4/16 (25%) patients. The combination of above normal threshold level in one sensor and below in the other is of particular importance because it carries the potential to change the clinical decision making process.

Determining the RAP every 4 min period (presently referred to as method 1) carries the potential risk of introducing high variability in the recorded value. In an attempt to reduce the variation in calculated RAP, some clinicians chose to use a moving window with updates every 5-10 seconds [18]. Incorporating such a moving time window with updates every 5-10 seconds means that the ‘oldest’ averaged value drops out and the ‘newest’ drops in, thereby slightly altering the Pearson correlation coefficient every 5-10 seconds. Presently we have shown that incorporating such a moving time window with frequent updates of RAP (method 2) do not reduce the discrepancy in RAP between the two signals, as compared to updating the RAP every 4-min period (method 1).

Another approach to reduce the variability in RAP is to average scores over a long time period, e.g. 20-30 min. However, averaging scores works as filtering of the calculated values, and has the drawback of hiding information. Averaging over long time periods will thus extensively mask differences in RAP. This can be seen in Table 2, left, where the average of RAP scores over many hours of recording reveals only minor differences in the RAP. From a clinical perspective, such multi-hour average values are of limited interest. Since RAP is recommended for use in the surveillance of critically ill patients, short-term updates of RAP would be needed. Averaging over 20-30 minute periods has hence less relevance since marked clinical deterioration may happen over such long periods.

Obviously, it can be disputed which differences in RAP that have clinical significance. In this context, it should be remembered that ICP and ICP-derived indices such as RAP are used in the surveillance of critically ill patients. An erroneous measurement revealed to the physician/nurse may represent potential harm to the patient. In this present study cohort, one ICP sensor showed RAP >0.6 while the other showed RAP <0.6 in about 1/10 observations (9-14%) in 8/16 patients. Given the diagnostic importance of such an index in patient management, we consider such a difference of clinical significance.

In the present patients, ICP monitoring was done as part of patient surveillance. Therefore, monitoring was independent whether the EVD was open or closed, and independent the opening pressure of the EVD. Since the ICP sensors were placed close by within the brain, both ICP sensors would be similarly impacted independent of drainage through the EVD.

The RAP should be considered together with the mean ICP since RAP may become de-coupled from the mean ICP when mean ICP is very high (>20-40 mmHg) [12]. It should be noted that mean ICP of the patients presented here was well below 20 mmHg, as recently reported for this same patient material [15].

### Baseline pressure errors (BPEs)

In 2006 Eide [13] described the phenomenon of baseline pressure errors (BPEs) when monitoring ICP simultaneously from two separate ICP sensors. The BPEs were manifested as marked differences in mean ICP combined with close to identity in ICP wave parameters such as the MWA [13]. In subsequent studies, BPEs could explain the differences in mean ICP observed when the two sensors were placed in different intracranial compartments [24,25]. By monitoring simultaneously from two separate ICP sensors, we have observed BPEs in solid sensors (Codman), air-pouch sensors (Spiegelberg) and fluid sensors (Edward’s Life science) [14]; indeed, BPEs have been observed in every type of ICP sensors tested, the Raumedic Neurovent P [26,27], the Codman [28], the Camino [29] and the Spiegelberg [30] sensor. Our recent prospective observational study has confirmed that BPEs occur frequently in the clinical setting, and can be of a magnitude that may affect clinical management [15]. BPEs may also explain the abrupt shifts and drifts in the relationship between mean ICP and MWA that are observed when monitoring using merely one ICP sensor [31].

In an experimental study, Eide and Bakken [32], showed that solid ICP sensors are sensitive to electrostatic discharges (ESDs) and observed BPEs in the form of sudden pressure shifts. Pressure drifts were thus seen following ESDs. The BPEs were of a magnitude that could alter patient management in a clinical setting given similar changes in mean ICP. This observation has recently been confirmed by others [33]. The cause of BPEs may be different when recording from a fiber-optic ICP sensor, a solid sensor based on the whetstone bridge principle, or from an air-pouch type of ICP sensor. All technical components of an ICP monitoring system (sensor, cable, transducer, display) represent potential sites of origin of BPEs. When monitoring ICP through an EVD, BPEs may in addition be created by imperfect fluid connection caused by air bubbles and debris, or through movement of the sensor position (height) relative to the measurement site [14].

### Impact of BPEs on ICP-derived indices

As illustrated in Figure 1, the BPEs were revealed as marked differences in mean ICP combined with close to identical ICP waveform. The largest differences in RAP were seen in patients 2, 3, 6, 7, 8, 9, 13, and 15 (Table 3). In these very same patients, there was also a high frequency and severity of BPEs.

Given that BPEs cause alterations in mean ICP, it is to be expected that every pressure index wherein the mean ICP is incorporated also becomes affected. However, the differences in RAP are less pronounced than the differences in mean ICP. This is because the RAP also incorporates the ICP amplitude, which is resistant to BPEs. For this reason, indices solely based on static pressure measures such as the pressure reactivity index (PRx), which is the moving correlation between mean ICP and mean arterial blood pressure (mean ABP) [34], can be anticipated to be particularly susceptible to BPEs.

Due to the effect of BPEs on the RAP indices, its feasibility as a guide in the management of neurosurgical patients will be hampered. In our opinion, the divergence in RAP from the two sensors is of such an extent that we do not advocate the use of RAP in clinical practice. In contrast, since the ICP wave amplitude is unaffected by BPEs, the ICP wave amplitude is a robust parameter. Thus, in a recent study comparing the ICP wave amplitude, ICP wave slope, and RAP as measures of intracranial compliance in head injury patients, the ICP wave amplitude was found to be superior to the other parameters [21].

## Conclusions

Simultaneous monitoring from two separate, close-by ICP sensors reveals significant differences in RAP that correspond to the occurrence of BPEs. As differences in RAP are of magnitudes that may alter patient management, we do not advocate the use of RAP as a guide in the management of neurosurgical patients.

## Abbreviations

BPE: Baseline Pressure Error; dP: Amplitude; dT: Rise time; dP/dT: Rise time coefficient; ESD: Electrostatic discharge; ICP: Intracranial pressure; MWA: Mean ICP wave amplitude; RAP: Correlation coefficient (R) between ICP wave amplitude (A) and ICP level (P); SAH: Subarachnoid haemorrhage; SW: Single wave; TBI: Traumatic brain injury.

## Competing interests

AS, TRM and WSO report no conflicts of interest. PKE has financial interest in the software company (dPCom A/S) that manufactures the software (Sensometrics® Software), which was used for digital recording of the continuous pressure signals in this study.

## Authors’ contributions

All authors contributed to conception and design, acquisition and interpretation of data. PKE contributed the bulk of the drafting of the manuscript and AS, TRM and WSO contributed with thorough editing of the manuscript. All authors have read and approved the final manuscript.
